# Hepatocyte growth factor levels in *Legionella *pneumonia: A retrospective study

**DOI:** 10.1186/1471-2334-11-74

**Published:** 2011-03-23

**Authors:** Futoshi Higa, Morikazu Akamine, Makoto Furugen, Kenji Hibiya, Michio Koide, Maki Tamayose, Yuichiro Tamaki, Syusaku Haranaga, Noriko Arakaki, Satomi Yara, Masao Tateyama, Jiro Fujita

**Affiliations:** 1 Department of Infectious, Respiratory, and Digestive Medicine, Control and Prevention of Infectious Diseases, Faculty of Medicine, University of the Ryukyus, Okinawa 903-0215, Japan

## Abstract

**Background:**

Hepatocyte growth factor (HGF) is known to be involved in the resolution of pulmonary inflammation and repair of acute lung injury. *Legionella *pneumonia is sometimes complicated by acute lung injury. Our study aimed to determine the role of serum HGF levels in *Legionella *pneumonia.

**Methods:**

Sera from patients with *Legionella *pneumonia (42 cases), other bacterial pneumonia (33 cases), pulmonary tuberculosis (19 cases), and normal controls (29 cases) were collected. The serum HGF levels for each serum sample were determined by sandwich ELISA. Clinical and laboratory data were collected by reviewing the medical charts.

**Results:**

Serum HGF levels were higher in patients with *Legionella *pneumonia than in those with other bacterial pneumonia, pulmonary tuberculosis, and controls. The HGF levels were compared with white blood cell counts, C-reactive protein, Alanine amino- transferase, and lactate dehydrogenase (LDH). The HGF levels were correlated to serum LDH levels. Moreover, serum HGF levels were significantly higher in non-survivors than in survivors.

**Conclusions:**

HGF levels increased in severer pneumonia caused by *Legionella*, suggesting that HGF might play a significant role in the *Legionella *pneumonia.

## Background

Hepatocyte growth factor (HGF) has been proved to be a multi-functional peptide growth factor, which plays important roles in lung development, lung inflammation, and repair [1]. Possible sources of HGF within the lung include bronchial epithelial cells, alveolar macrophages [2], and neutrophils [3]. The role of HGF in pulmonary infections has been studied in depth. Several studies have convincingly demonstrated that plasma/serum HGF levels become elevated in bacterial pneumonia [4,5]. Serum HGF levels are considered to be associated with the response of pneumonia to antimicrobials [6]. A study has previously shown that HGF is elevated in pulmonary fluid in acute lung injury (ALI) and acute respiratory distress syndrome (ARDS) and that higher HGF levels are associated with increased mortality [7].

*Legionella *spp. is a common causative pathogen of pneumonia, which can be fatal if complicated with acute lung injury (ALI) or acute respiratory distress syndrome (ARDS) [8]. The bacterial etiology of community-acquired pneumonia requiring ICU admission includes *Legionella *species as well as *Streptococcus pneumoniae*. However, the pathophysiology of fatal *Legionella *pneumonia remains unsolved. In this study, in order to clarify the role of HGF in *Legionella *pneumonia, the serum HGF concentrations in patients with *Legionella *pneumonia as well as other pulmonary infections were determined. We then evaluated the correlations between HGF levels and other clinical indicators and outcomes.

## Methods

### Study population

Our study included 42 consecutive cases of *Legionella *pneumonia diagnosed in our hospital. Further, 33 cases with other bacterial pneumonia, 19 cases with active pulmonary tuberculosis, and 29 age-adjusted control subjects were also included in this study. The pathogens isolated from the other bacterial pneumonia cases included *Streptococcus pneumoniae *(12 cases), *Hemophilus influenzae *(10 cases), *Klebsiella pneumoniae *(7 cases), *Mycoplasma pneumoniae *(4 cases), and others (3 cases). Control subjects were healthy without any infections, lung diseases, or liver diseases.

This study was approved by the University of the Ryukyus Institutional Review Board. The need for informed consent from each patient for inclusion in the study was waived because of the retrospective approach of this study, which caused no additional adverse events in any of the subjects. However, prior informed consent had been obtained from each patient before performing any procedure or obtaining any sample.

Blood samples were obtained for conventional clinical diagnosis from each patient. Bronchoalveolar fluids were obtained when required for the diagnosis of *Legionella *pneumonia. These samples were stored at -80°C until further use. Medical chart reviews were used to obtain information regarding the laboratory findings and clinical outcome of each patient.

### Diagnosis of pneumonia and tuberculosis

The diagnosis of pneumonia was based on the clinical presentation (symptoms and physical examination), chest X-ray findings, and laboratory data. The diagnosis of *Legionella *pneumonia was confirmed by the detection of *Legionella *by culture, elevation of antibody titers in paired sera, and/or detection of its specific antigen in the urine. The other pneumonia cases were diagnosed based upon the bacteriological investigations (blood culture and culture of the expectorated sputa with satisfactory quality for examination), pulmonary tuberculosis was diagnosed following a positive smear test for acid-fast bacilli as well as positive culture.

### HGF determination

Sera were prepared conventionally and stocked at -80°C until further investigation. The stock period was up to years without freeze-thaw cycle. The HGF level for each serum sample was determined by a sandwich ELISA (R&D Systems, Minneapolis, MN) using recombinant human HGF as a standard. The lowest detection limit for HGF was 40 pg/mL. This kit detects both active form and pro-form of HGF. Inter- and intra-assay reproducibilities are reported as 7.0% and 5.6%, respectively.

### Statistical methods

Data are reported as mean ± standard deviation (SD). The logarithmic transformation of several data values (HGF, white blood cell counts (WBC), and lactate dehydrogenase (LDH)) allowed Gaussian approximation (demonstrated by the Kolmogorov -Smirnov and Shapiro-Wilk tests). Differences in the logarithmically transformed values for HGF levels between multiple groups were examined by using analysis of variance (ANOVA) and Bonferroni's multiple comparison test. Differences between two groups were examined using unpaired *t*-test with Welch's correction and Mann-Whitney test. The relationship between two parameters was determined by Pearson's correlation coefficient test. These tests were performed using statistical software programs (Prism 4, Graphpad Software Inc., California; and SPSS version 15.0J, SPSS Japan Inc., Tokyo). *P *< 0.05 was considered statistically significant.

## Results

*Legionella *pneumonia cases were noted more frequently in males than in females; however, no difference was noted with respect to the mean age or rate of known liver disease as compared to other bacterial pneumonia cases (Table 1). Average time interval between onset to diagnosis (day of collecting samples) were 9.7 ± 5.8 days. Fourty-one among 42 *Legionella *pneumonia cases had prior treatment with beta-lactams. After establishment of diagnosis, macrolide, tetracycline, or quinolone antimicrobials were used. Single case had concomitant infection with *Enterococcus facium *and *Prevotella intermedia*, who was survivor. In this study, the mortality of *Legionella *pneumonia patients (19.0%) was higher than that for patients with other bacterial pneumonia (5.5%), but the difference was not statistically significant. Serum alanin aminotransferase (ALT) and LDH were higher in *Legionella *pneumonia cases than other bacterial pneumonia cases and pulmonary tuberculosis cases (Table 1).

**Table 1:** Characteristics of cases for this study

	*Legionella *pneumonia	Other bacterial pneumonia	Pulmonary tuberculosis	Control	*p*
cases	42	36	19	27	
age	58.9 + 12.0	58.3 + 18.2	56.7 + 16.4	54.2 + 19.1	n.s.
sex	37/ 5 ^a^	21 / 15	10 / 9	18 / 11	0.004 ^a^
outcome (alive/dead)	34 / 8	34 / 2	-	-	0.097
known liver disease	2 ^b^	2 ^b^	0	0	n. s. ^b^
ALT	66.4 + 58.7 ^c^	32.9 + 34.5 ^c^	21.2 + 16.2 ^c^	-	< 0.01 ^c^
LDH	807.3 + 748.9 ^d^	218.4 + 88.6 ^d^	239.1 + 302.4 ^d^	-	< 0.001 ^d^

a: M/F ratio was significant higher in *Legionella *pneumonia than in other bacterialpneumonia

b: n.s. not significant

c, d: ALT and LDH were significantly higher in *Legionella *pneumonia cases than in other bacterial pneumonia and pulmonary tuberculosis

**Figure 1:**
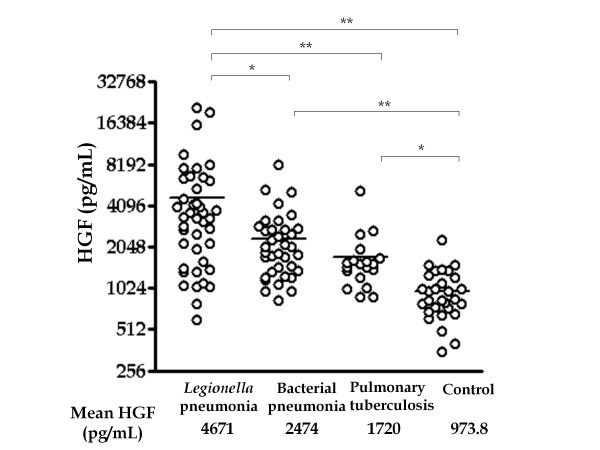
**Serum HGF levels in patients with *Legionella *pneumonia, other bacterial pneumonia, pulmonary tuberculosis, and controls**. Each symbol represents HGF levels at admission in each case. Analysis was performed by ANOVA and Bonferroni's test using normalized data by logarithmic transformation. *: *p *< 0.01, **: *p *< 0.001

Serum HGF levels were significantly elevated in *Legionella *pneumonia cases, other bacterial pneumonia cases, and pulmonary tuberculosis cases as compared to those in normal subjects (Figure 1). Further, the serum HGF levels were significantly higher in *Legionella *pneumonia cases than those in other bacterial pneumonia cases (Figure 1). To examine the influence of HGF levels by gender, the serum HGF levels in male and female patients of pneumonia and control subjects were compared; however, no significant differences were noted (data not shown). The male predominance of *Legionella *pneumonia did not influence the HGF levels.

**Figure 2:**
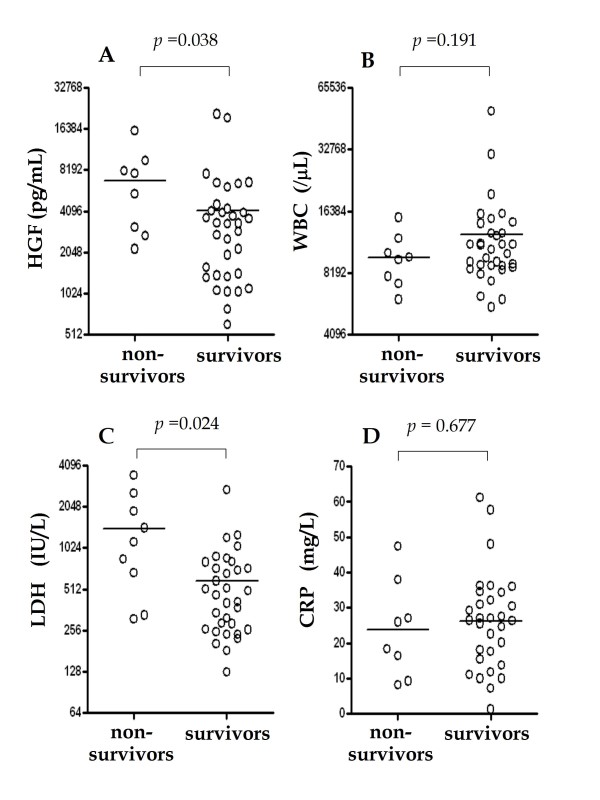
**Comparison of serum HGF and various bio-indicators (white blood cell counts, C-reactive protein, and LDH) in non-survivors and survivors of *Legionella *pneumonia**. Analysis was performed by the unpaired *t*-test with Welch's correction.

Next, serum HGF levels were compared with the clinical indicators of disease activity, i.e., WBC counts, C-reactive protein (CRP), PaO2/FiO2 ratio, LDH, and ALT levels (Table 2). Serum HGF levels were significantly correlated with LDH levels (Table 2). Serum HGF levels were not associated with the interval between onset of disease and the date when serum was obtained (data not shown). Extension of pulmonary lesion did not affect HGF levels. A comparison of serum HGF levels between survivors and non-survivors among *Legionella *pneumonia revealed that HGF levels and LDH activities of non-survivors were significantly higher than those of survivors (Figure 2), while WBC counts and CRP levels were not associated with the clinical outcomes (Figure 2). HGF levels in non-suvivors of other bacterial pneumonia (2 cases) were 5,183 pg/mL and 4,257 pg/mL. Among two cases of *Legionella *pneumonia with known liver diseases, one case was a non-survivor and HGF level was 9,584 pg/mL. Another case was a survivor and HGF level was 21,008 pg/mL.

**Table 2:** Association of HGF and other clinical indicators

biomarkers		correlation coefficient ( r )	r^2^	*p*^a^
HGF	WBC	0.207	0.043	0.195
HGF	CRP	0.201	0.040	0.226
HGF	PaO2/FiO2	-0.155	0.024	0.345
HGF	LDH	0.538	0.289	0.001
HGF	ALT	0.232	0.054	0.160
WBC	CRP	0.422	0.178	0.009
LDH	ALT	0.474	0.225	0.003

a: Association of Log(HGF) and each item was analyzed by Peason's coefficient test.

HGF: hepatocyte growth factor, WBC: white blood cell count, CRP: C-reactive protein, LDH: lactate dehydrogenase, ALT: alanine aminotransferase

## Discussion

Our study confirmed that serum HGF levels were elevated in pulmonary infections as reported previously [5,6,9]. However, our findings offered a new perspective by comparing serum HGF levels in pulmonary infections due to different pathogens. Serum HGF levels in patients with *Legionella *pneumonia were higher than in those with pneumonia caused by other pathogens. In this study, the differences in the clinical characteristics of *Legionella *pneumonia and other bacterial pneumonia pertained to mortality and the male/female ratio. The mortality noted for patients with *Legionella *pneumonia in this study (19.0%) was similar to that in previous reports, i.e., 5-20% [10-12]. The mortality noted for patients with other bacterial pneumonia in this study (5.5%) was similar to the report of a huge cohort study, i.e., 5.2% [13]. Therefore, the mortality for each group was considered typical. This study showed that HGF levels did not differ with gender. In present study, LDH and ALT activity was higher in *Legionella *pneumonia than other bacterial pneumonia and pulmonary tuberculosis. Higher LDH suggested severer organ damages and higher ALT suggested more frequent liver dysfunction. The factors influencing increased serum HGF levels in *Legionella *pneumonia might include severer form of the pneumonia and complication of liver dysfunction.

The results of previous studies evaluating the relationship between HGF levels and clinical outcomes seems to be controversial. One study demonstrated that serum HGF levels were lower in severe pneumonia than in non-severe pneumonia [14]. In the study, however, the cause of death include myocardial infarction (4 cases among 10 non-survivors). On the other hand, HGF levels in pulmonary edema fluids from non-survivors were noted to be higher than those in survivors of acute lung injuries [7]. Another study shows that serum HGF levels as a useful indicator of prognosis in inflammatory pulmonary diseases [15]. Present study revealed that in patients with *Legionella *pneumonia, serum HGF levels were higher in non-survivors than in survivors. Non-survivors were complicated by acute lung injury. Different cause of death might cause the discrepancy between several studies.

**Figure 3:**
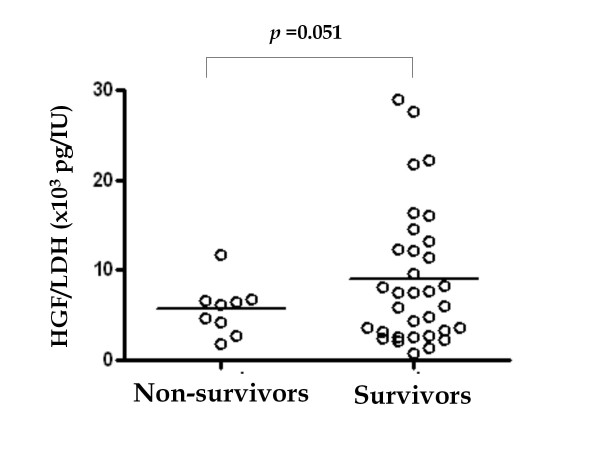
**Comparison of ratios of serum HGF and LDH activities in non-survivors and survivors of *Legionella *pneumonia**. Analysis was performed by the unpaired t-test with Welch's correction.

Serum HGF levels were correlated with LDH activity. The ratio of HGF/LDH seemed to be lower in non-survivors than in survivors (Figure 3). This finding did not achieve statistic significance, but our speculation based on this finding is that HGF works to repair the injury of lung and other organs, and insufficient HGF production in severe pneumonia might be associated with poor outcome. This would be worthwhile for further studies for confirmation. Other biomarkers (WBC counts and CRP) at the time of diagnosis were not associated with the clinical outcome.

## Conclusions

In conclusion, our study showed that HGF levels increased in severer pneumonia caused by *Legionella*, and suggested that HGF might play a significant role in the *Legionella *pneumonia. Further studies that investigate the precise role of HGF in severe *Legionella *pneumonia are warranted.

## Competing interests

The authors declare that they have no competing interests.

## Authors' contributions

FH was involved in design of this study, acquisition/analyses of data, and drafting the manuscript. MA, MF, KH, NA collected samples and carried out laboratory examinations. MK, MT, YT, SH, SY, and MT were involved in acquisition and analyses of data. JF was involved in design of study and preparing manuscript. All authors read and approved the final manuscript.

## Pre-publication history

The pre-publication history for this paper can be accessed here:

http://www.biomedcentral.com/1471-2334/11/74/prepub
